# Supplementary material for: Magnetic Field Effect in Bimolecular Rate Constant of Radical Recombination
Source: Int J Mol Sci. 2023 Apr 20;24(8):7555. doi: 10.3390/ijms24087555 (PMC10139179; doi:10.3390/ijms24087555)
Supplement: Supplementary file 1 [file ijms-24-07555-s001.zip › ijms-2339928-supplementary.pdf]

SUPPLEMENTARY MATERIALS

Magnetic field effect in bimolecular rate constant of radical recombination

Alexander B. Doktorov[1,2], Nikita N. Lukzen[1,3]

[1] International Tomography Center SB RAS,

630090, Novosibirsk, Russia

E-mail: luk@tomo.nsc.ru

[2] Institute of Chemical kinetics and Combustion SB RAS,

630090, Novosibirsk, Russia.

[3] Novosibirsk State University, 630090, Novosibirsk, Russia

Analytical calculation of the rate constant of singlet recombination in a strong magnetic field

For the calculation of the rate constant of recombination, one has to determine the time evolution of the collective spin density matrix of two radicals A and B situated in the "cage" of the solution. This evolution is determined by the Zeeman interaction of radical spins with the external magnetic field and with magnetic nuclei, longitudinal and transverse relaxation, by process of their recombination, and the relative motion of radicals. Radicals can end up in a solvent cage during encounter in solution or just be there at the initial instant of time. The calculations differ only by setting different initial conditions for the density matrix. This allows us to establish a connection between the kinetic characteristics of these two types of processes.

Let us consider spherically symmetric contact recombination, replacing the real exponential dependence of the recombination probability on the interradical distance r ($r = R$ is the closest approach distance) by a δ -shaped interaction

$$K_S(r) = K_S(r) = w_S \exp\left(\frac{r-R}{\Delta}\right) \to k_S \frac{\delta(r-R)}{4\pi r R}, \quad K_S(R) = w_S, \quad k_S = 4\pi R^2 w_S \Delta \qquad (1)$$

In this case, one obtains the following relation of the rate constant of the bulk reaction k with the value of the singlet recombination yield R_S^S of the geminate reaction for a contact singlet precursor

$$k = \frac{k_S}{4}(1 - R_S^S) \qquad (2)$$

The expression for the recombination yield R_S^S for contact singlet recombination and contact singlet precursor can be obtained from the general expression in Ref.[1] for molecular motion by continuous diffusion and has the following form: (3)

$$R_S = \frac{\bar{k}_S}{4+\bar{k}_S(1+c_T)}\left(1 + c_T + \frac{8(2(1+\alpha)+\bar{k}_S)}{\left(2\left(1+2\alpha+\sqrt{k_2^2+4q^2}\right)+(1+\alpha)\bar{k}_S\right)\left(4+\bar{k}_S(1+c_T)\right)+2\bar{k}_S(2(1+\alpha)+\bar{k}_S)}\right) \qquad (3)$$

Here we introduce the dimensionless reaction rate constant as well as the following dimensionless parameters

,

$$\alpha = \frac{1}{\sqrt{2}}\sqrt{\sqrt{k_2^2+4q^2}\ + k_2}, \quad c_T = \frac{1}{1+\sqrt{k_1}},$$
$$\bar{k}_S = \frac{k_S}{k_D} = w_S\frac{R\Delta}{D}, \qquad k_1 = \frac{R^2}{D}\left(\frac{1}{T_{1A}}+\frac{1}{T_{1B}}\right), \quad k_2 = \frac{R^2}{D}\left(\frac{1}{T_{2A}}+\frac{1}{T_{2B}}\right), \qquad q = \frac{R^2}{2D}(\omega_A-\omega_B) \tag{4}$$

These parameters are determined by the contact values of the Laplace transform of the non-stationary Green function $G_0(R,R,s)$ of free diffusion, through which the recombination efficiencies are expressed in [1], that is, it is used that:

$$4\pi RD\mathrm{Re}G_0\left(R,R;\ \frac{1}{T_{2A}}+\frac{1}{T_{2B}}-i(\omega_A-\omega_B)\right) = \frac{1+\alpha}{1+2\alpha+\sqrt{k_2^2+4q^2}},$$
$$\left|4\pi RDG_0\left(R,R;\ \frac{1}{T_{2A}}+\frac{1}{T_{2B}}-i(\omega_A-\omega_B)\right)\right|^2 = \frac{1}{1+2\alpha+\sqrt{k_2^2+4q^2}}, \tag{5}$$
$$c_T = 4\pi RDG_0\left(R,R;\ \frac{1}{T_{1A}}+\frac{1}{T_{1B}}\right) = \frac{1}{1+\sqrt{k_1}}$$

The calculation of the singlet recombination yield through the Green's function has the advantage compared to the calculation of the probability of singlet recombination in the diffusion motion of radicals, performed in [2], that allows us to obtain the result in a compact form and consider any type of molecular motion (i. e. not necessarily the diffusion of radicals).

In the case of diffusion-controlled recombination, i.e. when $k_S \to \infty$ from Eq. (2) and (3) one obtains:

.

$$k = \frac{k_D}{(1+c_T)}\left(1-\frac{2}{2+(1+\alpha)(1+c_T)}\right) \tag{6}$$

In the absence of relaxation and singlet-triplet mixing one has $c_T = 1, \quad \alpha = 0$ и and from Eq. (6), as it should be, follows that $k = \frac{1}{4}k_D = \pi RD.$

1. Doktorov, A. B. The influence of spin relaxation and locally strong spin exchange on magneto-spin effects in radical pairs in high magnetic fields. *The European Physical Journal Plus* **2021,** 136, (10), 992.
2. Mintz, R. G.; Pukhov, A. A. The influence of paramagnetic impurities on magnetic effects in radical reactions. *Chem. Phys.* **1985,** 87.
